# Changing trends in lipid profile and biomarkers of renal function and bone metabolism before and after switching from tenofovir disoproxil fumarate to tenofovir alafenamide: a prospective observational study

**DOI:** 10.1186/s12981-021-00354-y

**Published:** 2021-05-27

**Authors:** Mahoko Ikeda, Yoshitaka Wakabayashi, Koh Okamoto, Shintaro Yanagimoto, Shu Okugawa, Kyoji Moriya

**Affiliations:** 1 Department of Infection Control and Prevention, The University of Tokyo Hospital, 7-3-1, Hongo, Bunkyo-ku, Tokyo, Japan; 2 Department of Infectious Diseases, The University of Tokyo Hospital, 7-3-1, Hongo, Bunkyo-ku, Tokyo, Japan

**Keywords:** HIV-infection, Antiretrovirals, Biomarkers, Tenofovir disoproxil fumarate, Tenofovir alafenamide

## Abstract

**Background:**

Antiretrovirals, including tenofovir, can suppress human immunodeficiency virus (HIV) infection but cannot completely eradicate it. Patients with HIV infection are administered antiretroviral drugs over a long term; thus, managing consequent adverse drug reactions, such as renal dysfunction and bone mineral loss, is important. Currently, highly sensitive biomarkers that can detect adverse drug reactions early have not been well studied.

**Methods:**

This single-center, prospective, observational study explored changes in the biomarkers of renal function, bone metabolism, and lipid profile before and after switching from tenofovir disoproxil fumarate (TDF) to tenofovir alafenamide (TAF) in patients with HIV infection.

**Results:**

All 31 enrolled patients had been treated with antiretrovirals for more than 5 years. The rate of proteinuria decreased significantly after starting TAF-containing antiretroviral regimen. The urinary liver-type fatty acid binding protein (L-FABP)/creatinine ratio was significantly decreased at 3 and 6 months after switching to TAF compared with that before switching to TAF (− 0.5 μg/g Cr at 3 months, and − 0.8 μg/g Cr at 6 months; *p* < 005 for both at 3 and 6 months). The urinary N-terminal telopeptide (NTx)/creatinine ratio decreased over the study period, and the ratios were significantly different between 3 and 6 months (− 11 nmol/mmol Cr at 3 months, − 15.2 nmol/mmol Cr at 6 months; p = 0.0069 at 3 months, p < 0.0001 at 6 months). Low density lipoprotein-cholesterol level significantly increased at 3 (+ 26 mg/dL) and 6 months (+ 13 mg/dL) compared with that at the baseline (*p* < 0.0001).

**Conclusions:**

Switching from TDF to TAF decreased the levels of renal and bone biomarkers, such as urinary L-FABP and NTx, but increased low density lipoprotein-cholesterol levels. Future studies should evaluate if these biomarkers, such as urinary L-FABP and NTx, truly detect serious adverse drug reactions early.

## Background

Antiretrovirals (ARVs) have been successfully used to suppress human immunodeficiency virus (HIV) infection, and the life expectancy of the HIV-infected patients treated with ARVs has considerably increased, comparable to that of HIV-uninfected patients [1]. However, ARVs cannot completely eradicate HIV; therefore, lifelong therapy with ARVs is still required.

Long-term therapy with ARVs can cause adverse drug effects. Tenofovir disoproxil fumarate (TDF) was used since 2001 in the world and since 2004 in Japan. TDF has recommended as a part of initial regimens for most treatment naïve patients [2]. The adverse drug effects commonly caused by TDF include renal dysfunction and bone density loss [3]. Tenofovir alafenamide (TAF), a prodrug of tenofovir, was approved in Japan in 2017. TAF has improved permeability into the lympho-reticular cells and reduces the systemic concentration of tenofovir by 10% compared to TDF [4]. Clinical trials have shown that switching from TDF to TAF has improved the estimated glomerular filtration rate (eGFR) and levels of creatinine, urinary protein, and urinary biomarkers such as retinol-binding protein (RBP) [5, 6]. A pooled analysis of 26 clinical trials have also supported the improved renal safety of TAF compared to TDF [7].

Although switching to TAF from TDF showed renal improvement with potent viral suppression, highly sensitive biomarkers that indicate these subclinical changes after switching to TAF are not well-identified.

The urinary liver-type fatty acid binding protein (L-FABP) is expressed in the human proximal tubules, and its excretion via urine reflects tubulointerstitial damage [8]. L-FABP could be a prognostic biomarker for the development of renal dysfunction in HIV-infected patients [9].

Among the biomarkers of bone metabolism, bone-specific alkaline phosphatase (BAP) is a glycoprotein on the surface of osteoblasts that is released during bone formation. The N-terminal fragments of telopeptide (NTx) of type 1 collagen after degradation of the bone matrix is considered a resorption marker [10]. These markers have been evaluated in several studies [11–13] in patients on a TDF-containing regimen of antiretrovirals.

In this study, we aimed to explore the changing trends in biomarkers of renal function and bone metabolism, and lipid profile while switching from TDF- to TAF-containing regimen in HIV-infected patients.

## Methods

### Patients and setting

This observational study was conducted prospectively at the University of Tokyo Hospital, a 1217-bed tertiary-care teaching hospital in Tokyo, Japan. The study was approved by the research ethics committee of the Faculty of Medicine of the University of Tokyo. All the patients have provided written informed consent.

HIV-infected adult (20 years old and more) patients were enrolled in the study from July 2017 to June 2018. The enrollment criteria were as follows: (1) patients who had already received TDF-containing antiretroviral regimen for at least a year, (2) patients who could visit the outpatient clinic, and undergo blood and urine tests at least once every 3 months, and (3) if the physician had determined that the patients preferred TAF over TDF to be concerned about possible adverse drug reactions such as renal dysfunction and bone mineral loss for long treatment.

The study period of all the patients was for 6 months. Additional urine samples, and venous blood samples were collected for testing in a commercial laboratory during the study period.

### Data collection and selection

The collected patient data included age, sex, body weight, height, underlying disease (diabetes mellitus, hypertension, hyperlipidemia, chronic kidney disease (CKD), chronic hepatitis C virus infection, and chronic/history of hepatitis B virus infection), a history of acquired immunodeficiency syndrome (AIDS), and the prescribed antiretroviral regimen by medical chart review. Chronic kidney disease was defined as eGFR < 60 mL/min/1.73 m^2^ during the study period or previous diagnosis by their respective doctors. Body mass index was calculated as weight in kilograms divided by the square of height in meters: weight (kg)/height (m)^2^.

The following laboratory data were reviewed using the medical charts: CD4^+^ lymphocyte count, plasma HIV RNA level, complete blood count, serum phosphate level, low and high density lipoprotein cholesterol levels, triglyceride (TG) level, urinalysis, and serum creatinine level. Glomerular filtration rate (GFR) was calculated with serum creatinine and cystatin C levels.

In addition, serum and urine samples were tested for the following in a commercial laboratory (LSI Medience Corporation): urinary liver-type fatty acid binding protein level (U-L-FABP; latex agglutination immunoturbidimetric assay), urinary phosphate level, urinary *N*-acetyl-beta-d-glucosaminidase (U-NAG), cystatin-C (latex agglutination turbidimetry), urinary N-terminal telopeptide (U-NTx; enzyme immunoassay), and serum BAP (chemiluminescent enzyme immunoassay). Using this data the following creatinine ratios were calculated: U-L-FABP/creatinine (Cr), U-NAG/Cr, and U-NTx/Cr. To assess renal proximal tubular dysfunction, the rate of tubular reabsorption of phosphate (percent tubular reabsorption of phosphate; %TRP) was calculated according to the following formula:

$$\% \text{TRP} = \{ 1- (\text{urine phosphate
times serum creatinine)}/(\text{serum phosphate times urine creatinine})\}\times 100.$$

Proteinuria was defined by a value of ≥ 1+ by dipstick urinalysis.

### Statistical analysis

The Wilcoxon signed-rank test was performed using the JMP Pro software version 14.2.0 (SAS institute Inc., Cary, NC, USA) to analyze trends of biomarkers before and after switching from TDF to TAF. The significance level of the two-tailed test was set at 0.05.

## Results

Thirty-one patients were enrolled in our study. The patients’ characteristics are shown in Table 1. All the patients were men, and the median age was 45 years. The average BMI was 23.6, and 5 patients had low body weight (< 55 kg). Thirty patients were Japanese, and one was an African. All the patients had been treated with ARVs for more than 5 years. Half of the TDF-containing antiretroviral regimens also consisted of integrase inhibitors, such as dolutegravir and raltegravir. Among all the ARVs, only TDF was changed during the study period. Two patients in the study had baseline CKD. Eleven patients were diagnosed with hyperlipidemia, among which, two of them received statin treatment before switching to TAF.

**Table 1:** Patients’ characteristics

Patients’ characteristics			
Age: median (range)	45 (33–73)	Comorbidity	
Sex: male:female	31:0	Chronic kidney disease	2
BMI: average (range)	23. 6 (16.5–36.1)	Diabetes mellitus	2
History of AIDS	13	Hypertension	5
PCP	5	Hyperlipidemia	11
Lymphoma	2	HCV infection	3
CMV retinitis	2	HBV infection	20
Candida esophagitis	2	Antiretrovirals with tenofovir	
Kaposi’s sarcoma	1	DRV/r	10
Disseminated MAC	1	RAL	8
Cryptococcus meningitis	1	DTG	8
Duration of ART		EFV	4
Less than 5 years	11	FPV/r	1
5–10 years	19		
More than 10 years	1		
Nadir CD4^+^ T-cell count, (cells/μL)			
Less than 100	15		
100–199	6		
200–499	9		
Unknown	1		

*BMI* body mass index [calculated as weight in kilograms divided by the square of height in meters: weight (kg)/height (m^2^)], *AIDS* acquired immunodeficiency syndrome, *PCP* *Pneumocystis* pneumonia, *CMV* cytomegalovirus, *MAC* *Mycobacterium avium* complex, *HCV* hepatitis C virus, *HBV* hepatitis B virus, *DRV/r* ritonavir-boosted darunavir, *RAL* raltegravir, *DTG* dolutegravir, *EFV* efavirenz; *FPV/r* ritonavir-boosted fosamprenavir, *ART* antiretroviral therapy

There was no significant change in CD4^+^ T cell count, and a rate of successful viral suppression was maintained. The median CD4^+^ T cell counts were 448, 385, and 442 cells/µL at 0, 3, and 6 months, respectively, after switching from TDF to TAF. The rates of HIV RNA suppression (less than 100 copies/mL) were 93.5%, 90.3%, and 100% at 0, 3, and 6 months, respectively. All patients remained on ART during the study period. No events of bone fractures, acute coronary syndrome, and renal failure occurred.

### Biomarkers of renal function

The biomarkers of renal function such as the eGFR values calculated using creatinine, and with cystatin-C did not show any statistical change during the study period (Table 2). The rate of proteinuria decreased significantly after starting TAF-containing antiretroviral regimen.

**Table 2:** Analysis of serum and urine biomarkers before and after changing to tenofovir alafenamide among 31 patients

Median	Duration after switching			p value	
	0 months	3 months	6 months	3 months	6 months
Serum creatinine (mg/dL)	0.89 (0.68–1.16)	0.90 (0.66–1.11)	0.89 (0.6–1.25)	0.179	0.098
Estimated GFR using creatinine	73.1 (50.1–108.4)	72.6 (53.2–110.2)	74.8 (47.2–123.3)	0.238	0.098
Serum cystatin C (mg/L)	0.88 (0.65–1.39)	0.87 (0.64–1.26)	0.88 (0.62–1.26)	0.942	0.807
Estimated GFR using cystatin C	91.2 (50.2–130.5)	91.5 (56.4–131.6)	92.3 (56.4–136.2)	0.833	0.226
Urine-NAG/urine-creatinine (U/g Cr)	3.72 (0–56.9)	5.31 (0.83–28.6)	3.93 (0–29.4)	0.723	0.739
Urine-L-FABP/urine-creatinine (μg/g Cr)	2.3 (0.6–63.9)	1.8 (0.6–31.2)	1.5 (0.3–15)	0.0166*	0.0002*
%TRP	85.1 (73.7–92.7)	87.7 (73.6–97.7)	86.9 (74.2–94.2)	0.026*	0.200
Serum inorganic phosphorus (mg/dL)	3.1 (2.5–4.1)	3.0 (2.1–4.3)	3.1 (2.2–4.7)	0.992	0.841
Proteinuria (%)	13.3	6.67	6.67	0.023*	0.023*
Urine occult blood: positive (%)	6.7	6.7	10	0.573	0.973
Serum LDL-cholesterol (mg/dL)	106 (67–159)	132 (84–199)	119 (82–207)	< 0.0001*	< 0.0001*
Serum triglyceride (mg/dL)	144 (41–874)	146 (47–700)	175 (43–669)	0.192	0.273
Serum BAP (μg/L)	10.8 (7.0–21.6)	11.7 (6.2–21.1)	11.0 (7.4–19.8)	0.894	0.150
Urine NTx/urine Cr (nmol/mmol Cr)	48 (27.4–84.9)	37 (20.2–83.4)	32.8 (14.7–67.4)	0.0034*	< 0.0001*

*GFR* glomerular filtration rate, *NAG* *N*-acetyl-beta-d-glucosaminidase, *L-FABP* liver-type fatty acid binding protein, *TRP* tubular reabsorption of phosphate, *LDL* low density lipoprotein, *BAP* bone-specific alkaline phosphatase, *NTx* N-terminal fragments of telopeptide

*Urinalysis data were obtained from 30 of the 31 patients

**Fig. 1:**
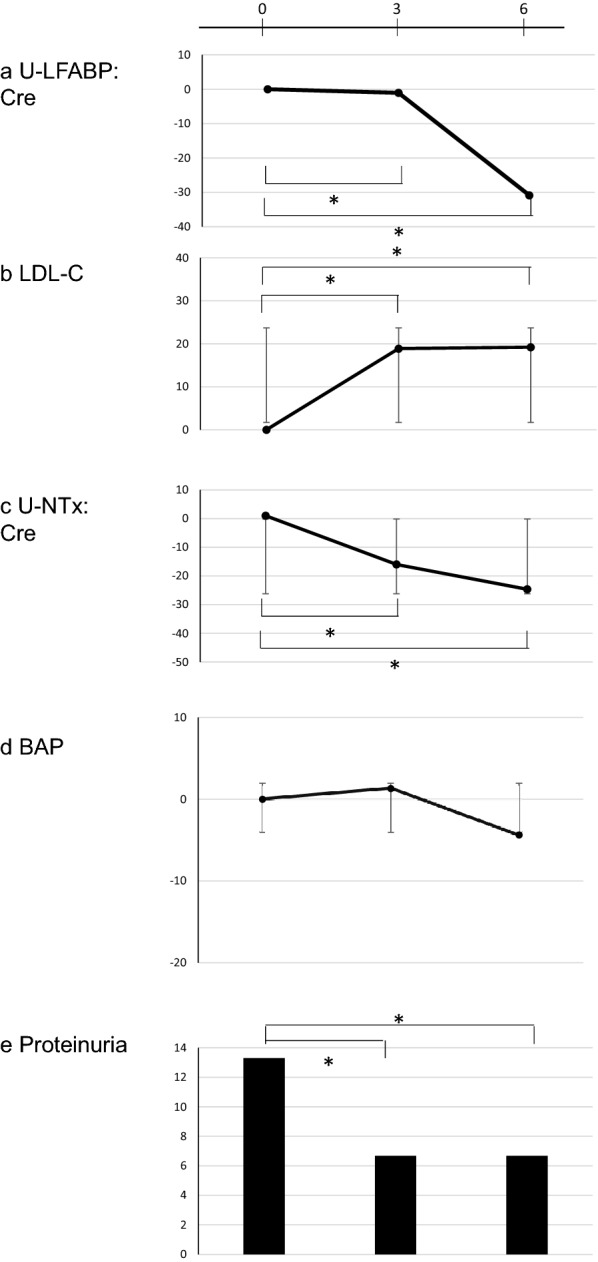
Rate of change in biomarkers before and after switching from tenofovir disoproxil to tenofovir alafenamide. Vertical axis represents the rate of change (%) of each biomarker compared to the baseline (before switching antiretrovirals). **a** Rate of change in urine-liver-type fatty acid binding protein/creatinine (L-FABP/Cr) levels. **b** Rate of change in low-density lipoprotein cholesterol (LDL-C) levels. **c** Rate of change in urine-N-terminal telopeptide/creatinine (U-NTx/Cr) levels. **d** Rate of change in bone-specific alkaline phosphatase (BAP) levels. **e** Percentage of detection of proteinuria

U-L-FABP/Cr ratios had significantly decreased at 3 and 6 months after switching to TAF, compared to the value before switching to TAF, as shown in Fig. 1a) (− 0.5 μg/g Cr at 3 months, and − 0.8 μg/g Cr at 6 months; *p* = 0.0166 at 3 months, *p* = 0.0002 at 6 months). U-NAG/Cr ratios did not show any statistical change during the study period.

The value of %TRP had significantly increased at 3 months (*p* = 0.026), but the rate of change in each patient was low.

### Lipid profile and biomarkers of bone metabolism

Low density lipoprotein-cholesterol (LDL-C) levels significantly increased at 3 months and 6 months compared to the baseline [+ 26 mg/dL at 3 months, and + 13 mg/dL at 6 months; *p* < 0.0001) (Fig. 1b)]. The significance was maintained regardless of co-administration with other ARVs, or hyperlipidemia at baseline.

BAP levels had not changed during the study period. The U-NTx/Cr ratios decreased during the study period, and there were statistical significances between all the points [− 11 nmol/mmol Cr at 3 months, − 15.2 nmol/mmol Cr at 6 months; p = 0.0069 at 3 months, p < 0.0001 at 6 months) (Fig. 1c)].

## Discussion

In this study, we reported the changes in biomarkers of renal function and bone metabolism, and lipid profile when switching from TDF to TAF in HIV-infected patients. During the study, all the patients continued the TAF-containing regimen of ARVs that showed potent viral suppression. There were no serious adverse effects in the first 6 months after switching to TAF-containing regimen.

There were no noticeable changes in the eGFR calculated using creatinine levels, and cystatin-C levels during the study period. The L-FABP levels and the rate of proteinuria were significantly decreased. These results might be subclinical for majority of the participants without having CKD. However, it has been reported that the renal function in Japanese HIV-infected patients decreased depending on the duration of treatment with TDF, especially in patients with low body weight [14]. Another observational study of a 12-year period also showed that decreased eGFR occurred after 3 months of TDF-containing regimen of ARVs, which was also related to low body weight [15].

Proteinuria is commonly observed in HIV-infected patients [16]. Its risk factors are exposure to TDF, older age, low CD4^+^ T-cell counts, anti-hepatitis C virus antibodies, and race [16–18]. Because the patients in our study were not older age and have already acquired high CD4^+^ T-cell counts by treatment including TDF on enrollment, major reason of the improvement of proteinuria might be discontinuation of TDF exposure. As Proteinuria itself is one of risk factors for CKD [19] and fragility fracture [17] in HIV-infected patients, early detection of proteinuria could motivate switching ARVs.

Because L-FABP was reported as a potential marker of CKD for patients without albuminuria [20], the detection of subclinical changes using urinary L-FABP prior to the progression to CKD is important in patients taking TAF, especially in those with low body weight.

%TRP change was observed at 3 months but not at 6 months. %TRP was a marker of renal proximal tubular dysfunction and calculated as value of one minus fractional excretion of phosphate. The fractional excretion of phosphate was reported as a possible marker of end-stage renal diseases such as dialysis in moderate to advanced CKD patients [21]. However, %TRP did not showed continuous change at 6 months after switching to TAF in our study. This result might because the difference of the patients’ background.

The elevation of biomarkers of lipids such as LDL-C was observed to have significance. Previous studies have also shown the elevation in the LDL-C and TG levels, but the range of elevation depended on the observational period, and the characteristics of the participants [22–24]. One of the major risks for hyperlipidemia that was identified after switching to TAF was the elevation at beginning in TG or LDL-C levels [25] although opposite results also reported [26]. In our study, LDL-C levels increased regardless of the hyperlipidemia at baseline. This risk population, that needs lipid-lowering drugs after switching TAF from TDF, must be extensively studied in the future.

Among the biomarkers of bone metabolism, U-NTx significantly decreased in our study. Previous study showed TDF-sparing regimen decreased the level of biomarkers of bone metabolism such as osteocalcin and bone alkaline phosphatase [13], and switching from TDF to TAF also improved bone mineral density [27]. Although it is unclear whether tenofovir directly influences osteoblasts, a change in gene expression was observed with tenofovir exposure in vitro [28]. Because U-NTx levels related bone mineral density levels in older men and women [29], U-NTx could also be used to predict current osteoporosis in TDF-exposure patients.

Here, the renal function and bone metabolism had improved within 6 months after switching to TAF. However, attention must be drawn to whether the lipid metabolism could be elevated up to the level that needs statin treatment.

According to patients’ genetic background [30, 31] the presence of various comorbidities, and body weight [14], clinicians might be required to determine whether to continue TDF or switch to TAF to prevent the progression to renal tubular dysfunction or metabolic disorders, using biomarkers such as urinary L-FABP and NTx. Its tailor-made design would help lives without disabilities of HIV-infected patients on ARVs.

Nevertheless, there were some limitations to our study. First, the information on the changes in lifestyle, such as new exercise habits and dietary patterns, were not collected using a questionnaire, although the treating physicians had recorded the changes in lifestyle and adherence to ARVs during each visit and no other drugs was changed. Second, the study did not have long observational period. Finally, this study was a single-centered observational study, and all except one was Japanese.

## Conclusions

The TAF-containing regimen of ARVs could be safe with potent viral suppression for 6 months after switching from a TDF-containing regimen of ARVs. Switching from TDF to TAF decreased the levels of biomarkers such as urinary L-FABP and NTx but increased LDL-C levels. Future studies should evaluate if these biomarkers truly detect serious adverse drug reactions early.

## Data Availability

The datasets used and analyzed during the current study are available from the corresponding author upon reasonable request.

## Abbreviations

HIV: Human immunodeficiency virus
ARV: Antiretrovirals
TDF: Tenofovir disoproxil fumarate
TAF: Tenofovir alafenamide
eGFR: Estimated glomerular filtration rate
RBP: Retinol-binding protein
L-FABP: Liver-type fatty acid binding protein
BAP: Bone-specific alkaline phosphatase
NTx: N-terminal fragments of telopeptide
CKD: Chronic kidney disease
AIDS: Acquired immunodeficiency syndrome
TG: Triglyceride
CrCl: Creatinine clearance
NAG: *N*-Acetyl-beta-d-glucosaminidase
TRP: Tubular reabsorption of phosphate
BMI: Body mass index
LDL-C: Low density lipoprotein-cholesterol
PCP: Pneumocystis pneumonia
CMV: Cytomegalovirus
MAC: Mycobacterium avium complex
HCV: Hepatitis C virus
HBV: Hepatitis B virus
DRV/r: Ritonavir-boosted darunavir
RAL: Raltegravir
DTG: Dolutegravir
EFV: Efavirenz
FPV/r: Ritonavir-boosted fosamprenavir
ART: Antiretroviral therapy
